# A Microcapsule‐Integrated smRandom‐seq Platform Enables Fixation‐Free Single Microbe RNA Sequencing

**DOI:** 10.1002/advs.77013

**Published:** 2026-08-05

**Authors:** Mengdi Song, Yuting Wang, Zhaolun Wang, Shunji Zhang, Zhengmin Tang, Yongcheng Wang

**Affiliations:** ^1^ Department of Laboratory Medicine of the First Affiliated Hospital & Liangzhu Laboratory Zhejiang University School of Medicine Hangzhou China; ^2^ M20 Genomics Hangzhou China; ^3^ College of Biomedical Engineering and Instrument Science Zhejiang University Hangzhou China

**Keywords:** aqueous two‐phase system, fixation‐free workflow, microcapsule, single‐microbe RNA sequencing

## Abstract

Single‐microbe RNA sequencing (smRNA‐seq) emerges as a powerful approach for resolving microbial heterogeneity at single‐cell resolution, but current high‐throughput workflows still depend on chemical fixation to preserve cell‐of‐origin transcriptomic information during lysis, permeabilization, and downstream processing. Although fixation is integral to current smRNA‐seq workflows, its broader impact on overall performance remains unresolved because a practical alternative is lacking. Here, we present an smRNA‐seq strategy that integrates aqueous two‐phase system microcapsules with our previously developed smRandom‐seq method. Using polyethylene glycol diacrylate‐ and dextran‐based microcapsules, our method physically confines bacterial cells and released nucleic acids within individual microcompartments, thereby enabling single‐cell transcript capture in both fixation‐based and fixation‐free workflows. With two model species, *Escherichia coli* and *Acinetobacter baumannii*, we show that fixation‐free processing improves smRNA‐seq performance, increasing per‐cell gene detection sensitivity by approximately 50% relative to fixed controls while preserving similar overall transcriptional profiles. To our knowledge, this microcapsule‐integrated smRandom‐seq platform achieves the highest transcriptomic capture sensitivity among current high‐throughput smRNA‐seq platforms. This framework provides a practical alternative to fixation in high‐throughput smRNA‐seq and opens new prospects for the development of single‐microbe omics technologies.

## Graphical abstract

We developed a microcapsule‐integrated high‐throughput fixation‐free single‐microbe RNA‐seq workflow in which microbes are encapsulated in aqueous two‐phase microcapsules for physical confinement of nucleic acids. This platform enables co‐encapsulation with barcoded beads for sequencing and reveals that fixation‐free processing enhances transcriptomic capture sensitivity, achieving approximately a 50% increase in median gene detection relative to fixed controls.

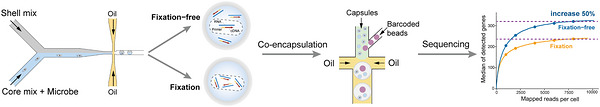

## 1 Introduction

Microbes inhabit nearly all ecological niches on Earth, with heterogeneous microbial communities driving vital environmental processes, including nutrient cycling, decomposition, and primary production [1, 2]. Beyond these ecological roles, microbes exploit their functional diversity to contribute significantly to bio‐industries [3] and regulate human health, both as commensal microbiota and as etiological agents of infectious diseases [4, 5, 6]. As listed in one of Nature's “Seven technologies to watch in 2025” [7], recent advances in high‐throughput single‐microbe RNA sequencing (smRNA‐seq) technologies are providing unprecedented insight into complex microbial populations. High‐throughput smRNA‐seq enables transcriptomic profiling of thousands of cells at single‐cell resolution, driving the identification of rare subpopulations in genetically identical populations [8, 9], the reconstruction of transient regulatory states [9], and the characterization of transcriptional dynamics associated with antibiotic tolerance [10], virulence activation [11], metabolic specialization [12], and stress adaptation [13].

Current high‐throughput smRNA‐seq approaches largely fall into two platform strategies, split‐pool and droplet‐based methods. Split‐pool methods such as PETRI‐seq [8] and microSPLiT [9] iteratively barcode cells across microwells to enable scalable profiling, whereas droplet‐based methods including ProBac‐seq [14], BacDrop [10], M3‐seq [15], and smRandom‐seq [16] encapsulate individual cells with barcoded primers in microfluidic droplets for high‐throughput profiling. Despite these implementation differences, both strategies converge on a common workflow in which cell fixation remains essential for preserving cell‐of‐origin transcriptomic information throughout processing. In practice, microbial samples for smRNA‐seq are typically pre‐fixed to cross‐link transcripts to intracellular proteins and limit RNA leakage during permeabilization, lysis, and repeated washing and handling steps, thereby reducing RNA diffusion and ambient RNA–driven cross‐cell contamination. Although widely used during sample processing, the inherently chemical nature of fixation may influence smRNA‐seq outcomes through altered molecular accessibility and shifts in reaction efficiency. Since no currently established high‐throughput platform enables a complete workflow on fixation‐free microbial cells, direct evaluation of how fixation influences transcriptomic outcomes has remained difficult.

Recently, the introduction of aqueous two‐phase system (ATPS) microcapsules into eukaryotic single‐cell RNA‐seq has provided a new perspective on this challenge by enabling physical confinement of cells and released nucleic acids during downstream processing [17, 18]. This strategy suggests a potential route toward fixation‐free smRNA‐seq, in which transcript retention is achieved through compartmentalization rather than chemical cross‐linking. Here, we introduce a microcapsule‐integrated smRNA‐seq method adapted from smRandom‐seq that enables single‐cell isolation, lysis, and in vivo‐compatible transcript capture directly from fixation‐free bacterial cells. We achieved the fabrication of polyethylene glycol diacrylate‐ and dextran‐based microcapsules at the 20 µm scale, enabling the confinement of single microbes within physical microcompartments to preserve cell‐of‐origin transcriptomic information. Coupled with on‐capsule reactions, our approach eliminates the requirement for paraformaldehyde fixation and enables direct transcriptomic profiling of viable bacteria. Using this adapted platform, and with capsule–barcoded bead co‐encapsulation rates exceeding 70%, we evaluated the effect of fixation on high‐throughput smRNA‐seq performance in *Escherichia coli* and *Acinetobacter baumannii*. Notably, we observe that fixation‐free processing yields an approximately 50% increase in the median number of genes detected relative to fixed controls and maintains similar overall transcriptional profiles across the two conditions. To our knowledge, this fixation‐free smRNA‐seq platform achieves the highest median gene number among current high‐throughput smRNA‐seq platforms. Together, this methodology establishes a new framework for high‐throughput smRNA‐seq that enables high‐resolution interrogation of microbial physiology in fixation‐free cells and expands single‐cell analysis to experimental settings in which chemical fixation cannot be readily applied.

## 2 Results

### 2.1 Single‐Microbe ATPS Microcapsule Generation and Integration Into Droplet‐Based smRNA‐seq Pipeline

An ATPS is a water–water phase‐separated mixture in which a stable interface forms between two immiscible aqueous phases generated by combining incompatible hydrophilic components [19, 20]. Here, we adapted ATPS to encapsulate bacterial cells in discrete aqueous microcapsules prior to cell processing in smRNA‐seq, creating a physical barrier that confines cells and their released nucleic acids while remaining fully compatible with downstream enzymatic reactions.

**FIGURE 1:**
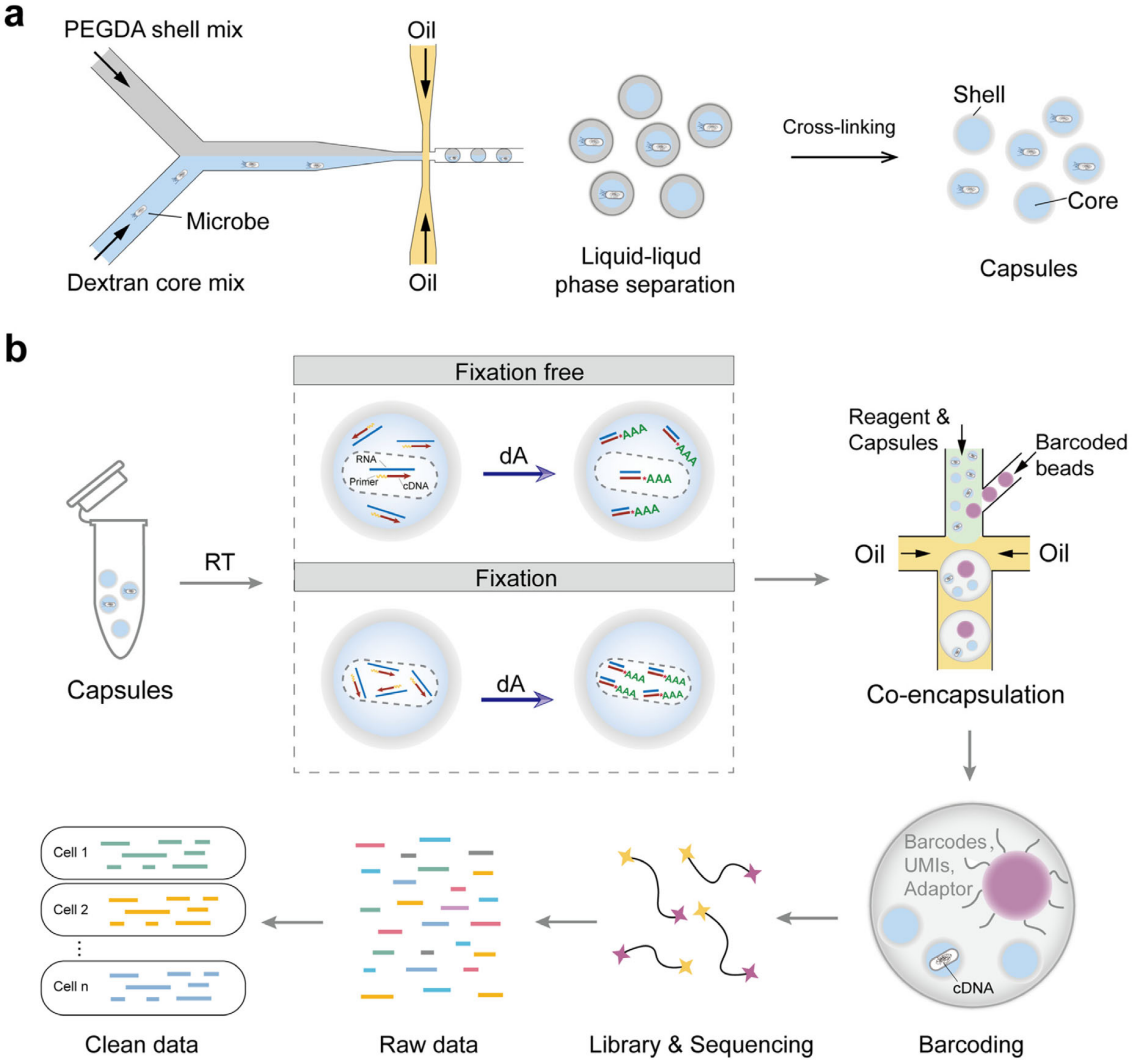
Microfluidic generation of microbial microcapsules and integration into a droplet‐based smRNA‐seq pipeline. (a) Schematic of the capsule‐generation chip for co‐flow of core mix, shell mix, and oil to produce uniform microbial microcapsules after cross‐linking. (b) Overview of downstream microcapsule processing, including lysis, split reverse transcription, pooling, polyA tailing, co‐encapsulation with barcoded beads, sequencing library preparation, and processing of sequencing data.

We designed a microcapsule‐generation chip comprising three inlets for the oil phase, the core mix, and the shell mix, together with a single outlet for high‐throughput droplet generation (Figure S1). In this device, the core phase with microbes is enveloped by the shell phase and segmented by the oil flow to generate uniform single‐cell compartments (Figure 1a), which form stable microcapsules after cross‐linking of the shell phase. We then integrated these microcapsules into our previously developed droplet‐based smRandom‐seq pipeline. Briefly, microcapsules are permeabilized using detergent and lysozyme, followed by split reverse transcription with pre‐indexed random primers and pooling for polyA tailing, and are subsequently co‐encapsulated with barcoded beads, enabling cDNA extension within droplets prior to polymerase chain reaction (PCR) amplification, purification, and library preparation for sequencing (Figure 1b).

### 2.2 Optimization of ATPS Microcapsule Composition and Flow Conditions

**FIGURE 2:**
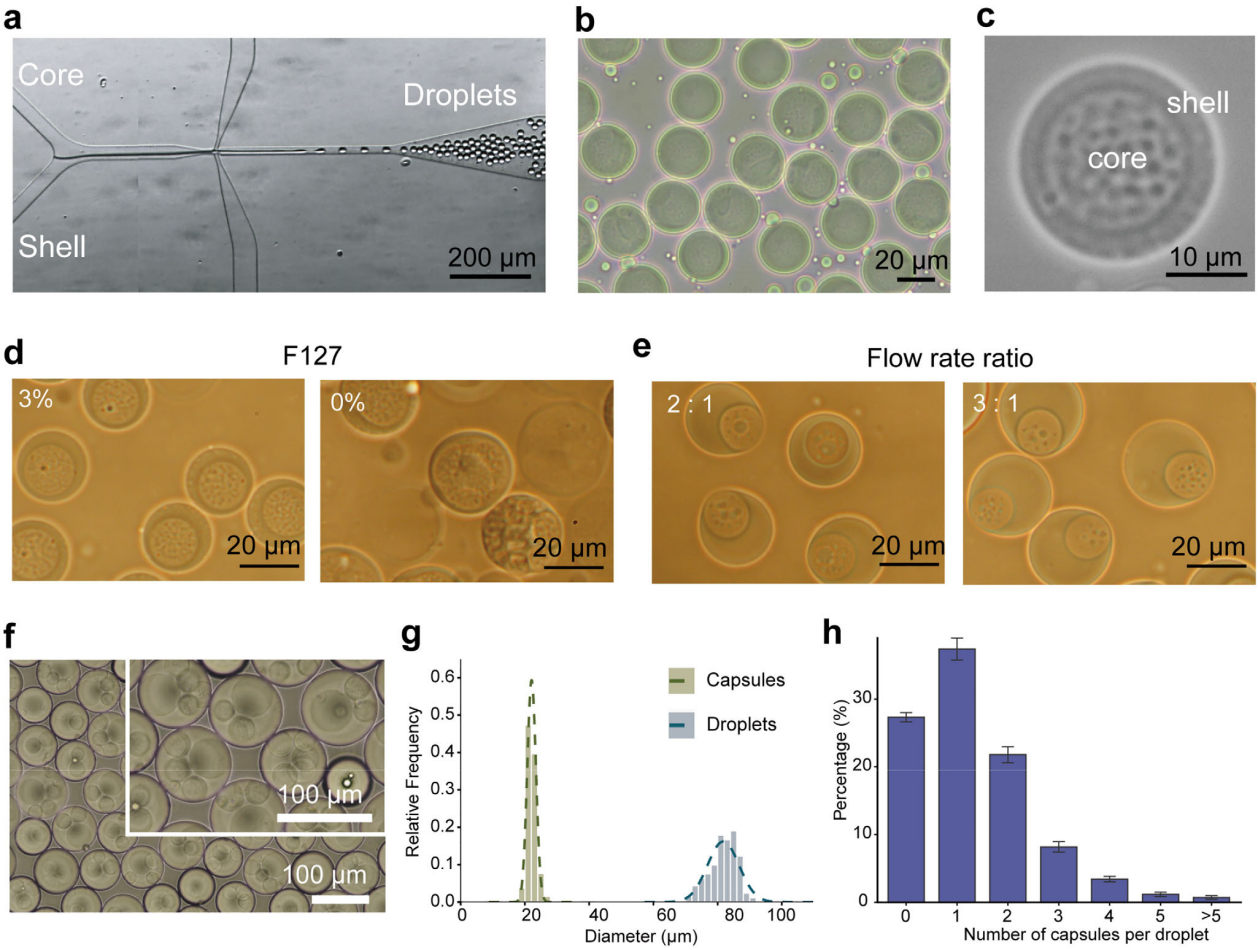
Optimization of ATPS microcapsule formation and co‐encapsulation for downstream droplet processing. (a) Microfluidic generation of aqueous two‐phase system (ATPS) droplets in the device. (b) Representative image showing a uniform population of ATPS droplets produced using the optimized formulation. (c) Bright‐field image of a microcapsule with a clear core–shell architecture. (d) Effect of F127 on microcapsule morphology, showing that inclusion of F127 preserves a sharp shell–core interface, whereas its exclusion disrupts phase separation. (e) Effect of shell‐to‐core flow rate ratio on microcapsule concentricity, with increased ratios shifting the core toward the microcapsule edge. (f) Microfluidic co‐encapsulation of microcapsules with barcoded beads for downstream droplet‐based processing. (g) Size distributions of microcapsules and co‐encapsulating droplets, showing that most microcapsules measured 19–24 µm in diameter and that droplets were highly uniform and centered around 80 µm. (h) Microcapsule occupancy distribution per droplet, showing that most droplets contained one or two microcapsules and that the overall capsule–barcoded bead co‐encapsulation rate reached 70%, supporting stable and efficient loading for downstream single‐droplet processing applications.

To optimize ATPS microcapsule production and RNA retention performance, we iteratively screened shell and core formulations and converged on a balanced composition that supports microcapsule mechanical stability and selective molecular permeability. The optimized shell phase consisted of 9% polyethylene glycol diacrylate (PEGDA) 5000, 9% PEGDA 400, 3% F127, and 1.5% ammonium persulfate (APS), in which longer PEGDA 5000 supports efficient phase separation and shorter PEGDA 400 increases shell rigidity through a higher cross‐link density (Figure S2). Complementing this shell formulation, the core phase comprised 9% dextran supplemented with 0.1% F68 to ensure reliable partitioning relative to the PEGDA‐rich shell phase while maintaining biocompatibility with bacterial cells. To further evaluate the compatibility of F127 and F68 with microbial membrane integrity, we performed a co‐culturing test of *E. coli* with F127 and F68 at workflow‐relevant concentrations and observed normal bacterial growth over time (Figure S3). With this formulation, we generated uniform ATPS droplets in a microfluidic device (Figure 2a,b) and obtained microcapsules with a clear core–shell architecture (Figure 2c). Notably, the inclusion of F127 in the shell phase was essential for maintaining a sharp shell–core interface, as its exclusion disrupted microcapsule morphology and weakened phase separation (Figure 2d). We next tuned the flow conditions of the shell and core phases and found that microcapsule concentricity was maximized at a 1:1 shell‐to‐core ratio, whereas higher ratios of 2:1 or 3:1 shifted the core toward the microcapsule edge (Figure 2e). A markedly off‐center core would create locally uneven shell thickness, which would compromise capsule recovery during emulsion breaking and washing. Finally, we demonstrated co‐encapsulation of microcapsules with barcoded beads to interface with downstream droplet processing (Figure 2f and Figure S4). With most microcapsules measuring 19–24 µm in diameter, the co‐encapsulating droplets were also highly uniform and centered around 80 µm (Figure 2g). Together with the microcapsule occupancy profile, in which most droplets contained one or two microcapsules and an overall capsule–barcoded bead co‐encapsulation rate of 70% (Figure 2h), these results demonstrate stable and efficient capsule–bead co‐encapsulation for downstream single‐droplet processing applications.

### 2.3 Fixation‐Free ATPS Microcapsules Improve Nucleic Acid Release and cDNA Yield in Droplet‐Based smRNA‐seq

Next, we used two widely used bacterial models, *E. coli* and *A. baumannii*, to evaluate the performance of microcapsule‐integrated smRNA‐seq and to assess the impact of fixation on downstream readouts. Microbial cultures were combined into a mixed sample and encapsulated into ATPS microcapsules, and parallel samples were handled either fixation‐free with immediate lysis or paraformaldehyde‐fixed prior to lysis and downstream processing.

**FIGURE 3:**
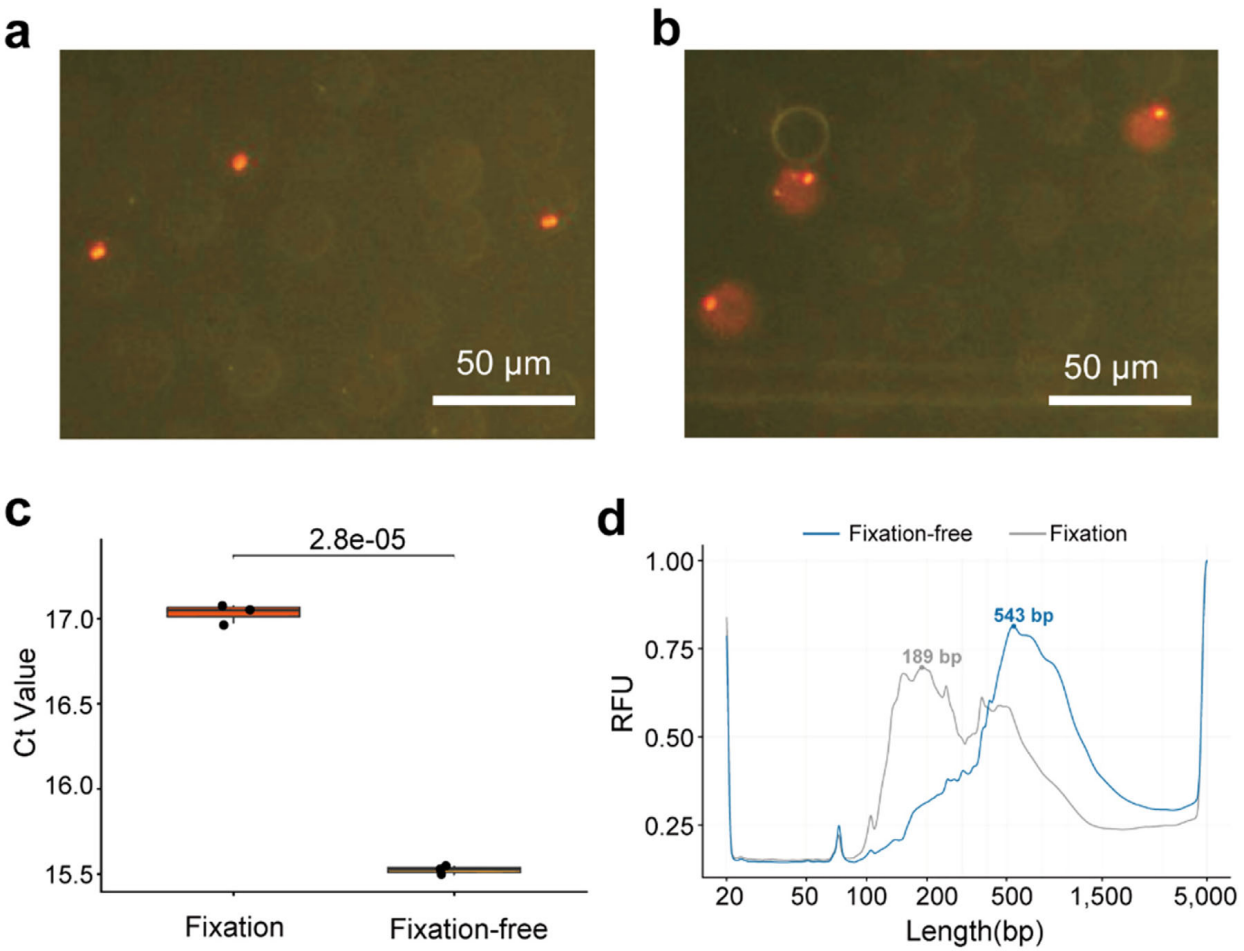
Fixation‐free microcapsule processing improves nucleic‐acid release and cDNA recovery from encapsulated bacteria. (a) Fluorescence microscopy of fixed samples stained with propidium iodide after microcapsule encapsulation and permeabilization, showing nucleic‐acid signal retained within rod‐shaped bacterial cell bodies in the microcapsule core. (b) Fluorescence microscopy of fixation‐free samples stained with propidium iodide, showing rod‐shaped bacterial cells together with diffuse nucleic‐acid signal distributed throughout the microcapsule core, consistent with nucleic‐acid release in the absence of fixation. (c) Comparison of qPCR Ct values between fixation‐free and fixed samples, showing significantly lower Ct values for fixation‐free samples across technical replicates, consistent with higher cDNA yield and improved amplification efficiency. (d) Fragment analysis of amplified cDNA from fixation‐free and fixed samples, showing a right‐shifted size distribution in fixation‐free sample relative to fixed sample, indicative of enhanced recovery and a larger cDNA product profile.

Following microcapsule encapsulation and permeabilization, fluorescence microscopy with propidium iodide revealed distinct intracellular and extracellular nucleic‐acid patterns between conditions. Fixed samples retained clear rod‐shaped bacterial signals confined to the cell bodies within the microcapsule core (Figure 3a), whereas fixation‐free samples showed rod‐shaped cells accompanied by diffuse nucleic‐acid signal throughout the microcapsule core (Figure 3b). This microscopy observation suggests that, without fixation, nucleic acids are released from permeabilized cells yet remain physically retained within the microcapsule compartment for capture. To further assess whether released nucleic acids could be retained by the PEGDA capsule shell, we performed a permeability assay using fluorescent nucleic acid probes. The 23 bp probe readily diffused into the capsule interior, whereas the 670 bp probe showed minimal entry, indicating that the PEGDA shell permits diffusion of very small nucleic acid species but substantially restricts larger nucleic acid molecules in the several‐hundred‐base‐pair range (Figure S5). We next quantified cDNA yield and amplification efficiency under both conditions. Compared with fixed cells, fixation‐free samples exhibited higher qPCR fluorescence signals across cycles and correspondingly lower Ct values (Figure S6). Technical replicates also showed a significant difference between groups (Figure 3c), suggesting increased amounts of recoverable nucleic acids in fixation‐free samples. Consistent with enhanced recovery and improved cDNA integrity, fragment analysis further showed a right‐shifted cDNA size distribution for fixation‐free sample relative to fixed sample, indicating an overall larger cDNA product profile (Figure 3d).

### 2.4 Benchmarking Microcapsule‐Based smRNA‐seq at the Sequencing Level

**FIGURE 4:**
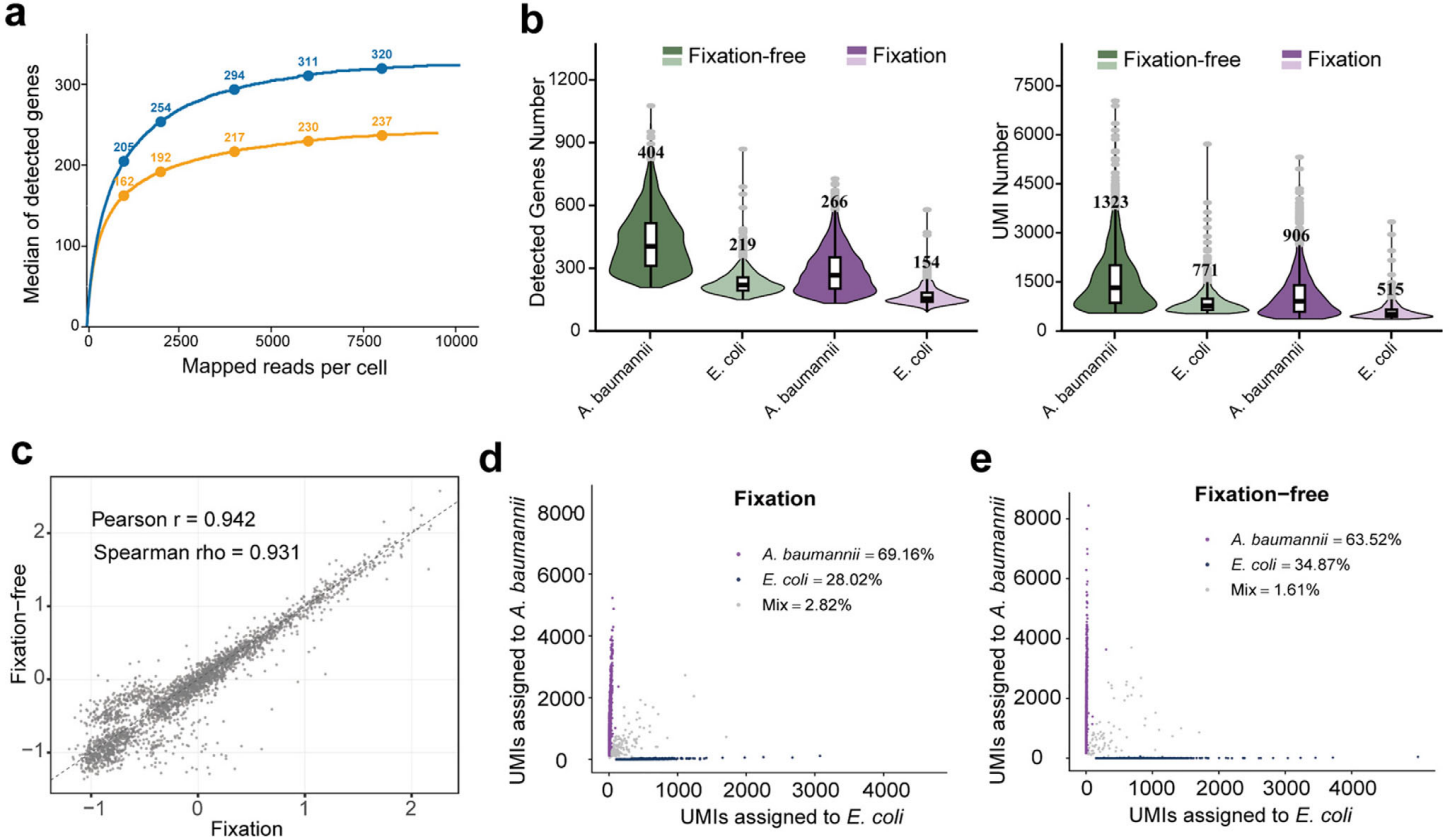
Fixation‐free microcapsule processing improves transcript detection while preserving global expression structure and maintaining low cross‐contamination. (a) Sequencing saturation analysis based on read subsampling per barcode. In both fixation and fixation‐free conditions, the median number of detected genes increased with mapped reads per cell and then approached a plateau. Across the full sequencing‐depth range, the fixation‐free condition consistently remained above the fixed condition, indicating higher transcript detection at matched read depth. (b) Violin plots showing distributions of detected gene numbers per barcode (left) and UMI counts per barcode (right) for *A. baumannii* and *E. coli* under fixation‐free and fixed conditions. Median detected gene numbers increased from 266 to 404 in *A. baumannii* and from 154 to 219 in *E. coli* under fixation‐free processing. Median UMI counts increased from 906 to 1323 in *A. baumannii* and from 515 to 771 in *E. coli*. These upward shifts indicate improved transcript recovery in the absence of fixation. (c) Scatter plot comparing per‐gene average expression across shared genes between fixation‐free and fixed samples. Expression profiles were highly concordant, with Pearson and Spearman correlation coefficients of 0.942 and 0.931, respectively, indicating that fixation‐free processing preserves the overall transcriptional landscape despite improved sensitivity. (d, e) Crosstalk analysis of mixed‐species single‐cell libraries under fixed (d) and fixation‐free (e) conditions. Each point represents a barcode plotted by UMI counts assigned to *E. coli* and *A. baumannii*. Most barcodes were assigned predominantly to a single species, with only a small fraction showing mixed species reads. The mixed‐barcode fraction was 2.82% in the fixed sample and 1.61% in the fixation‐free sample, indicating that omission of fixation does not measurably increase barcode mixing or sample‐to‐sample contamination.

With the single‐cell sequencing data, we performed saturation analysis by subsampling reads per barcode. In both conditions, the median number of detected genes increased with mapped reads per cell and then approached a plateau but remained consistently higher in the fixation‐free condition across the full sequencing‐depth range (Figure 4a). Consistent with this depth‐normalized trend, the distributions of the numbers of detected genes and unique molecular identifier (UMI) counts per barcode were also shifted upward in the fixation‐free condition for both species (Figure 4b), indicating that the increase in gene detection is not solely attributable to deeper sequencing but instead reflects improved effective transcript recovery. For both *A. baumannii* and *E. coli*, fixation‐free processing showed a clear upward shift relative to fixation, with the median number of detected genes increasing from 266 to 404 in *A. baumannii* and from 154 to 219 in *E. coli* (Figure 4b, left) (Figure S7). UMI distributions showed that median UMI counts increased from 906 to 1323 in *A. baumannii* and from 515 to 771 in *E. coli*, further supporting improved transcript capture without fixation (Figure 4b, right).

To evaluate whether the increased cDNA yield and gene detection could be affected by genomic DNA‐derived products, we examined the genomic annotation distribution of mapped reads. Intergenic reads accounted for only 0.67% of reads in the fixation‐free sample and 1.15% in the fixed control, whereas genic reads accounted for 99.33% and 98.85%, respectively. These results indicate that genomic DNA had a limited impact on the observed improvement. Next, to determine whether the selective permeability of the microcapsule shell affected transcript recovery by gene length, we analyzed gene detection across the 0–1000 bp range. Although the shortest gene‐length bin showed slightly lower detection, the length‐binned detection profiles were highly consistent between fixation and fixation‐free workflows for both species. Importantly, fixation‐free processing generally showed higher gene detection across the examined length range (Figure S8). These results suggest that potential diffusion of very short nucleic acid species does not substantially reshape the overall gene‐length‐dependent detection profile.

To evaluate whether fixation‐free processing altered the overall transcriptional landscape, we compared per‐gene average expression across shared genes between fixation‐free and fixed samples. This analysis showed high concordance, with Pearson and Spearman correlations of 0.942 and 0.931, respectively (Figure 4c). We next examined whether the improved gene detection in the fixation‐free workflow reflected procedure‐associated stress responses. Gene‐level differential detection analysis showed that genes preferentially detected in the fixation‐free sample were broadly distributed across functional categories, including metabolism and energy production, gene expression and genome maintenance, transport and membrane functions, and other cellular processes (Figure S9). Stress‐response genes represented only a small fraction of fixation‐free‐preferentially detected genes and were not significantly enriched compared with the remaining analyzed genes by Fisher's exact test (OR = 0.92, p = 0.69). Collectively, these findings indicate that fixation‐free processing maintains global expression profiles while improving per‐cell detection sensitivity.

Crosstalk analysis revealed similarly low cross‐contamination in both conditions, with only a small fraction of barcodes showing evidence of mixed‐species reads (Figure 4d,e). Specifically, the mixed‐barcode fraction was 1.61% in the fixation‐free sample and 2.82% in the fixed sample, indicating that the absence of fixation does not measurably increase barcode mixing or sample‐to‐sample contamination in this microcapsule‐adapted smRNA‐seq workflow. We next estimated the doublet rate from the mixed‐species experiment using standard barnyard logic. In such experiments, the observed mixed‐species fraction represents only interspecies doublets, whereas same‐species doublets are not detectable in the barnyard plot. Under the usual assumption that the two species are mixed at similar proportions and have similar probabilities of encapsulation, the total doublet rate can be approximated as twice the observed mixed‐species fraction. Based on this calculation, the estimated doublet rate for the fixation‐free workflow was approximately 3.2%.

Finally, we evaluated the sequencing performance of the fixation‐free smRandom‐seq workflow in the context of representative high‐throughput smRNA‐seq platforms, as summarized in Table 1. Among these smRNA‐seq platforms, the fixation‐free smRandom‐seq exhibited the highest transcriptomic capture sensitivity, whereas reported values from other platforms ranged from 43 to 371 detected features per cell, depending on the platform, species, and metric used. We next reviewed the sensitivity metrics at similar sequencing depths across platforms. At comparable sequencing depths, microSPLiT [9] reported 25 median genes per *E. coli* cell at approximately 4000 reads per cell, whereas the fixation‐free workflow detected approximately 216 median genes per cell at the same mapped‐read depth. ProBac [11] reported 165 median genes per cell at 1070 reads per cell, compared with approximately 175 median genes per cell for the fixation‐free workflow at a similar mapped‐read depth. M3‐seq [15] reported approximately 120 median genes per cell for the highest *E. coli* MG1655 rRNA‐depleted subgroup at approximately 4000 reads per cell, whereas the fixation‐free workflow detected approximately 219 median genes per cell at the same depth. However, such comparisons remain challenging since published datasets differ substantially in sequencing depth, capture chemistry, rRNA depletion strategy, species, and bioinformatic pipelines.

**TABLE 1:** Representative high‐throughput smRNA‐seq platforms, barcoding strategies, and transcriptomic capture sensitivity.

smRNA‐seq platform	Cell Barcoding Approach	Species	Transcriptomic Capture Sensitivity
PETRI‐seq [8]	Split‐pool	*E. coli*	103 median operons	
		*S. aureus*	43 median mRNA UMIs	
microSPLiT [9]	Split‐pool	*B. subtilis*	230 median genes	
		*E. coli*	138 median genes	
ProBac‐seq [14]	Droplet	*E. coli*	165 median genes	
		*B. subtilis*	241 median genes	
		*C. perfringens*	153–215 median transcripts	
BacDrop [10]	Droplet	*E. coli*	90 average genes	
		*K. pneumoniae*	88 average genes	
M3‐seq [15]	Droplet	*B. subtilis*	371 median genes	
		*E. coli*	151 median genes	
Fixation‐free smRandom‐seq	Droplet	*E. coli*	219 median genes	
		*A. baumannii*	404 median genes	

## 3 Discussion

This work presents an smRNA‐seq workflow that enables transcriptomic profiling of fixation‐free microbial samples. In conventional high‐throughput smRNA‐seq workflows, fixation serves as a critical measure for maintaining accurate single‐cell transcript assignment, whereas its omission may compromise transcript compartmentalization and increase ambient contamination during multistep processing. However, previous studies have shown that formaldehyde fixation forms reversible hydroxymethyl adducts on RNA bases and methylene‐bridge cross‐links between RNA and nearby biomolecules [21, 22]. These modifications can reduce RNA accessibility and interfere with primer annealing and elongation during reverse transcription, with greater impairment potentially occurring for longer RNA fragments or with excessive fixation [23, 24]. By contrast, PEGDA–dextran ATPS microcapsules confine individual microbes and their released nucleic acids within an aqueous core and permit reagent access through a semipermeable shell. This physical compartmentalization substitutes for chemical cross‐linking and enables fixation to be omitted while maintaining single‐cell transcriptome integrity. By optimizing shell and core compositions as well as microfluidic flow parameters, we fabricated PEGDA–dextran microcapsules with stable core–shell architecture, high concentricity, and durable mechanical integrity. Using the model species *E. coli* and *A. baumannii* encapsulated in these optimized microcapsules, we validated this ATPS microcapsule–integrated smRandom‐seq platform. Furthermore, we evaluated the effect of fixation on smRNA‐seq performance and transcript detection, an assessment that was not feasible with previously developed microbial smRNA‐seq platforms. Notably, the fixation‐free sample yielded higher RNA‐derived cDNA recovery and showed higher gene detection sensitivity, as well as exhibiting a transcriptional profile highly similar to that of the fixed sample. In addition, the fixation‐free workflow maintained similarly low barcode cross‐contamination relative to the fixed control. These findings collectively indicate that microcapsule‐enabled fixation‐free smRNA‐seq processing markedly outperforms the fixed control and exhibits superior performance relative to existing smRNA‐seq methods. The improved performance likely arose from the shift from cross‐linking–based immobilization to physical confinement of intracellular contents, which preserved the free diffusion of nucleic acids and other reaction‐relevant biomolecules within an accessible liquid core and thereby promoted a more homogeneous reaction environment than that produced by chemical fixation. Nevertheless, the selective permeability of the microcapsules could be further tailored through refinement of the formulation and generation process to achieve an application‐specific balance between nucleic acid retention and enzymatic accessibility. Such refinement may involve systematic experimental evaluation of parameters related to polymer formulation, cross‐linking conditions, and phase‐separation characteristics. As an alternative, sequential droplet merging represents a potential fixation‐free strategy in which cell lysis, reverse transcription, and barcoding are performed in separately generated droplets that are combined stepwise [25]. However, its implementation as an alternative to fixation in bacterial smRNA‐seq would require precise synchronization and one‐to‐one droplet matching, while limited opportunities for intermediate washing could introduce reagent carryover and reaction‐compatibility constraints. Together, our study supports ATPS microcapsules as a practical alternative to fixation for smRNA‐seq.

Though solely *A. baumannii* and *E. coli* were evaluated as model species in this study, the broader potential of this platform likely extends well beyond these organisms. By physically confining released nucleic acids within an individual microcompartment, the microcapsule design may be particularly advantageous for hard‐to‐lyse bacteria with unusual cell‐envelope architectures, such as Mycobacterium and Staphylococcus [26, 27, 28]. In such contexts, microcapsule‐based confinement could enable enhanced lysis while minimizing the contamination risk typically associated with transcript diffusion under over‐lysis conditions. This capability could be particularly valuable for infectious disease research, where single‐microbe transcriptomic analysis of pathogens causing tuberculosis and skin infections could reveal rare cell states linked to persistence, virulence, and antibiotic resistance [29, 30, 31, 32, 33]. Furthermore, the biocompatible nature of the microcapsules suggests additional experimental flexibility beyond transcript capture alone. Previous studies of microcapsule systems have shown compatibility with incubation‐based workflows [17], raising the possibility of coupling viable bacterial single‐cell transcriptomics with perturbation assays such as drug treatment and stress exposure. In addition to supporting fixation‐free smRNA‐seq, our study may provide a basis for extending microcapsule‐based workflows to other single‐microbe omics applications, including multimodal strategies such as combined RNA and DNA sequencing, by permitting sequential reagent access and supporting stepwise processing of omics assays within the same confined microcompartment. Taken together, these features highlight microcapsule‐integrated smRNA‐seq not only as a fixation‐free alternative for improved transcript recovery, but also as a versatile framework for extending single‐cell microbial research to more challenging species, multimodal omics applications, and dynamic functional studies.

## 4 Conclusions

Our study establishes ATPS microcapsules as a fixation‐free interface between viable bacteria and droplet‐based smRNA‐seq. Our comparison of fixation‐free and fixed conditions showed that substituting physical confinement for chemical fixation improved cDNA yield and size distribution, increased gene detection sensitivity, and maintained low cross‐contamination in smRNA‐seq. This framework has the potential to expand the experimental space for microbial single‐cell transcriptomics and provide a foundation for diverse applications across microbial research.

## 5 Methods

### 5.1 Bacterial Culture

*Escherichia coli* BW25113 and *Acinetobacter baumannii* ATCC17978 were provided by the First Affiliated Hospital, Zhejiang University School of Medicine. Both bacteria were cultured in Luria‐Bertani (LB) medium at 37°C with shaking at 220 rpm.

### 5.2 Bacteria‐Encapsulated ATPS Microcapsule Generation

All reagents used for capsule generation were pre‐incubated under ice‐cold conditions, and the overall capsule generation procedure was completed within approximately 20 min to limit potential procedure‐associated transcriptional changes. Based on the optimal cell‐loading conditions established in our previous study [34], the core phase solution was prepared by diluting cultured bacterial cells into 1X Dulbecco's phosphate‐buffered saline (DPBS) (Sigma, D8537) containing 9% dextran (MCE, HY‐112624P) and 0.1% F68 (Gibco, 24040032) to reach an optimal concentration of 20 000 cells per µL. The shell phase solution contained 9% PEGDA 5000 (Adamas, 43183JD), 9% PEGDA 400 (Adamas, 43183CG), 3% F127 (MCE, HY‐158231), and 1.5% APS (Sangon Biotech, A100486‐0025) in 1X DPBS. The oil phase solution was prepared by dissolving 0.5% TEMED (Thermo Fisher, 17919) in carrier oil (008‐FluoroSurfactant‐2wtH‐50G). Microcapsules were generated using a microfluidic chip with a height of 20 µm, with flow rates of 90 µL/h for the core phase, 90 µL/h for the shell phase, and 500 µL/h for the oil phase. ATPS droplets were collected in a 1.5 mL tube and immediately cross‐linked by incubation at 37°C for 20 min. After incubation, ATPS droplets were broken and purified by washing twice with 20% 1H,1H,2H,2H‐perfluoro‐1‐octanol (PFO) (vol/vol) in HFE‐7500 oil and then with HFE‐7500 oil. The purified microcapsules were resuspended in 1X DPBS supplemented with 0.1 U/µL RNase inhibitor (RI) (Thermo Fisher, N8080119)) and were ready for downstream application.

### 5.3 Bacteria‐Encapsulated ATPS Microcapsule Permeabilization

ATPS microcapsules were pelleted by centrifugation and resuspended in 1 mL pre‐chilled 100 mm Tris–HCl (pH 7) supplemented with RI, then centrifuged at 4000 g for 1 min at 4°C. The pellet was resuspended in 250 µL pre‐chilled 0.04% Tween‐20 (Sangon Biotech, A600560) in PBS and permeabilized on ice for 3 min. Microcapsules were then washed twice by centrifugation at 4000 g for 1 min at 4°C, after which cells were counted. Microcapsules containing approximately 50 million cells were collected and resuspended in 147.5 µL of nuclease‐free water. For cell wall digestion, reactions were assembled with 147.5 µL of the microcapsule suspension, 40 µL Lyso‐Buffer, 2.5 µL RNase inhibitor, and 10 µL lysozyme. All reagents were provided in the VITApilote‐PFT1200 kit (M20 Genomics, R20115124). Microcapsules were incubated at 37°C for 15 min in a thermocycler, then quenched by adding 1 mL pre‐chilled PBS‐RI. Microcapsules were washed twice by centrifugation at 4000 g for 1 min at 4°C and finally resuspended in 1 mL pre‐chilled PBS‐RI.

### 5.4 Pre‐Barcoding Reverse Transcription

Reverse transcription was performed with VITApilote‐PFT1200 kit (M20 Genomics, R20114124). Microcapsules were evenly aliquoted into 14 PCR tubes, and a distinct pre‐barcoded random primer was added to each tube. Each reaction contained 2.25 µL of cells in PBS, 0.25 µL 100 mm dNTPs, 1 µL 5 × reverse transcription buffer, 0.25 µL RNase inhibitor, 0.25 µL reverse transcriptase, and 1 µL 10 µm pre‐barcoded random primer. All reagents and primers were provided in the VITApilote‐PFT1200 kit. Reactions were run with 12 cycles of multiple annealing using a ramp from 8°C to 42°C, followed by incubation at 42°C for 30 min.

### 5.5 dA Tailing

dA tailing was performed with VITApilote‐PFT1200 kit (M20 Genomics, R20114124). After reverse transcription, 1 µL of 50 mm EDTA was added to each tube to stop the reaction. Microcapsules from all tubes were then pooled into a single PCR tube and washed three times to remove residual reagents and unincorporated random primers. For dA tailing, reactions were assembled with 39 µL of cells in PBS, 5 µL buffer T1, 5 µL buffer T2, 0.5 µL TT enzyme, and 0.5 µL 100 mm dATP, followed by incubation at 37°C for 30 min. All reagents were provided in the VITApilote‐PFT1200 kit.

### 5.6 ATPS Microcapsule Barcoding

Based on the concentration of cells in microcapsules, microcapsules were diluted in a density gradient solution. Microcapsules, 2 × DNA extension reaction mix, and ready‐to‐use hydrogel barcoded beads were then co‐encapsulated into droplets using the VITAcruizer DP400 microfluidic platform (M20 Genomics, E20000131) with the corresponding chip (M20 Genomics, E20000131). Reagents required for droplet generation were supplied in the VITApilote‐PFT1200 kit. Droplets were subsequently incubated at 37°C for 1 h to release the barcoding primers from the beads and promote primer hybridization, at 50°C for 30 min to activate the warm‐start polymerase and initiate barcoded second‐strand synthesis, at 60°C for 30 min to enhance polymerase activity, reduce template secondary structure, and complete double‐stranded DNA synthesis, and at 75°C for 20 min to heat‐inactivate the enzymes.

### 5.7 ATPS Microcapsule Dissolution and Single‐Cell Library Preparation

After the extension reaction, microcapsules were released from droplets using perfluorooctane (Sigma, 370533). Microcapsules were then dissolved in 1 m NaOH and incubated at 40°C for 20 min. The reaction was neutralized with 1 m acetic acid and 50 mm Tris–HCl. The aqueous phase was purified using AMPure XP beads (Beckman Coulter, A63881), and the recovered cDNA was PCR‐amplified, followed by a second AMPure XP cleanup and elution in nuclease‐free water. All required reagents and primers were provided in the VITApilote‐PFT1200 kit. Purified cDNA was quantified using a Qubit 4.0 fluorometer (Invitrogen, Q33238) and size‐profiled on a 4200 TapeStation (Agilent, G2991BA).

Libraries were constructed using the VAHTS Universal DNA Library Prep Kit for Illumina V3 (Vazyme, ND607‐03/04). Fragmented cDNA was subjected to end repair and A‐tailing by incubating a reaction containing 50 ng cDNA, end‐repair buffer, end‐repair enzymes, and nuclease‐free water at 30°C for 30 min, followed by heat inactivation at 65°C for 30 min. The product was then ligated to adapters by adding ligation enzymes and a working adaptor and incubating at 20°C for 15 min. Ligated DNA was purified using AMPure XP beads, followed by library amplification and a second purification. Final libraries were quantified using a Qubit 4.0 fluorometer and size‐profiled on a 4200 TapeStation. Sequencing was performed on a NovaSeq 6000 using an S4 Reagent Kit with 150‐bp paired‐end reads.

### 5.8 Fluorescent Nucleic Acid Probe Permeability Assay

Purified ATPS microcapsules were incubated with fluorescent nucleic acid probes of different sizes. Two probe sizes were tested: a short 23 bp fluorescent nucleic acid probe and a longer 670 bp fluorescent nucleic acid probe. Briefly, microcapsules were resuspended in buffer containing each fluorescent probe and incubated under the same solution conditions used for downstream capsule handling. After incubation, the capsules were imaged by fluorescence microscopy. Probe entry into the capsule interior was assessed by comparing the fluorescence signal inside the capsule core with the surrounding background.

### 5.9 Culturing Test with F127 and F68 Surfactants

*E. coli* cells were cultured in the presence of the surfactants at the concentrations used in the microcapsule workflow, including 3% F127 and 0.1% F68. Bacterial cells were monitored by bright‐field microscopy at 0, 3, and 6 h after the start of incubation.

### 5.10 Single‐Cell RNA Sequencing Data Analysis

Raw sequencing data were preprocessed by removing primer sequences and trimming poly(dA) tails. For each Read1, the 8‐nt UMI and the 20‐nt cell barcode were identified and extracted. Sequenced barcodes were corrected by merging reads that could be uniquely assigned to an accepted barcode within a Hamming distance of ≤2. Read2 was aligned to a combined reference genome comprising *E. coli* (GCF_000750555.1) and *A. baumannii* (GCF_902728005.1) using STAR (v2.7.10a), retaining only uniquely mapped reads to generate the gene expression matrix.

Downstream analysis was performed in Seurat (v4.3.0). The expression matrix was imported to construct a Seurat object, retaining genes detected in at least one cell. Data were normalized using NormalizeData, and the 6,000 most variable genes were identified with FindVariableFeatures. These genes were z‐scored using ScaleData and subjected to principal component analysis for dimensionality reduction. Cells were clustered using FindNeighbors and FindClusters based on PCs 1–15. Clusters were visualized by UMAP using a minimum distance of 0.3 and a resolution of 0.03. Differentially expressed genes were identified with FindAllMarkers to support cluster annotation, using average log2 fold change as an effect‐size metric.

### 5.11 Sequencing Saturation Analysis

Saturation curves were generated by plotting mapped reads per cell against the median number of detected genes per cell for each condition. To enable direct comparison at comparable sequencing depths, data points nearest to predefined target read depths (1000, 2000, 4000, 6000, and 8000 reads per cell) were identified separately for each sample and highlighted on the plot. The corresponding median gene counts were annotated directly on the figure.

### 5.12 Gene‐Level Expression Concordance Analysis

Gene‐level expression profiles between the fixation and fixation‐free single‐cell datasets were compared using Seurat objects in R. Raw RNA count matrices and normalized RNA expression matrices were extracted from each object, and only genes shared between the two datasets were included in the analysis. For each shared gene, the number and proportion of cells with non‐zero counts were calculated in each sample, and only genes detected in at least one cell in both datasets were retained for downstream analysis. Mean gene expression was then calculated across all cells using the normalized RNA assay data after inverse log‐transformation (expm1) of values stored in the Seurat data slot. For each gene, the log2 fold change in fixation‐free relative to fixation was calculated, and genes were classified as fixation‐free‐biased (log2FC ≥ 1), fixation‐biased (log2FC ≤ ‐1), or balanced. Global concordance between the two datasets was assessed by calculating Pearson and Spearman correlations based on log10‐transformed mean expression values, with a small pseudocount added to avoid undefined values. The results were visualized as a scatter plot of average gene expression in the two datasets, with a diagonal reference line indicating equal expression and correlation coefficients annotated on the figure.

### 5.13 Cross‐Species Transcript Crosstalk Analysis

Cross‐species transcript crosstalk analysis was conducted using Seurat objects in R for the fixation and fixation‐free datasets. Raw RNA count matrices were extracted, and genes were assigned to either the BW25113 or J7R21 reference based on gene name prefixes. For each cell, UMIs assigned to BW25113 and J7R21 were quantified by summing counts across all genes associated with each reference, and the total assigned UMI count was calculated as the sum of these two values. Cells with no assigned UMIs were excluded. The fraction of BW25113‐derived UMIs was then calculated for each cell, and cells were classified as BW25113 if at least 90% of assigned UMIs originated from BW25113, as J7R21 if at least 90% originated from J7R21, or as mixed otherwise. The proportions of BW25113, J7R21, and mixed cells were calculated for each dataset. Crosstalk was visualized using scatter plots of *E. coli*‐assigned vs. *A. baumannii*‐assigned UMI counts per cell, with cells colored by classification and axis ranges harmonized across conditions for direct comparison.

### 5.14 Gene‐Level Differential Detection, Functional Annotation, Stress‐Response Assessment, and Gene‐Length‐Binned Analysis

Gene‐level differential detection was performed using filtered gene‐barcode matrices from the Fixation and Fixation‐free workflows. For each gene, barcodes with nonzero counts were defined as detected, and detection rates were calculated as the percentage of detected barcodes in each sample. Differential detection was assessed using Fisher's exact test based on detected and non‐detected barcode counts, followed by multiple‐testing correction to obtain false discovery rate values. The detection‐rate difference was calculated as the Fixation‐free detection rate minus the Fixation detection rate. Genes were classified as higher in Fixation‐free, higher in Fixation, or not significant. Pseudobulk gene counts were generated by summing counts across barcodes, followed by calculation of species‐level counts per million (CPM) values and log2 CPM ratios between Fixation‐free and Fixation. Stress‐response annotations were added using a curated stress‐response gene list, and functional annotations were grouped into major categories. For functional analysis, differentially detected genes were grouped by workflow direction and major functional category, and gene counts and within‐group percentages were calculated. Stress‐response enrichment among Fixation‐free‐high genes was tested using Fisher's exact test by comparing annotated stress‐response genes in the Fixation‐free‐high gene set with the remaining analyzed genes.

Gene‐length‐binned detection analysis was performed using filtered Matrix Market files from the Fixation sample and Fixation‐free sample. Genes within 0–1000 bp were assigned to species‐specific bins: 41–200, 203–398, 401–599, 602–800, and 802–1000 bp for *E. coli*; and 62–200, 203–398, 401–599, 602–800, and 803–998 bp for *A. baumannii*. For each species and length bin, genes with counts greater than zero were considered detected, and detected genes per barcode were calculated using column‐wise sums of nonzero entries. Detection distributions were shown as violin plots with median crossbars. The percentage of detected genes per barcode was calculated as detected genes divided by total genes in the corresponding bin multiplied by 100. Median detection percentages were then calculated for each species, workflow, and bin, paired between Fixation and Fixation‐free samples, and compared using Pearson and Spearman correlations with linear regression trend lines.

## Author Contributions

**Zhaolun Wang**: software, visualization. **Mengdi Song**: investigation, validation, formal analysis, writing – original draft, writing – review and editing, data curation, visualization, methodology, conceptualization. **Yuting Wang**: methodology, validation, visualization, writing – review and editing. **Yongcheng Wang**: methodology, validation, visualization, writing – review and editing. **Zhengmin Tang**: resources, conceptualization, validation. **Shunji Zhang**: validation, visualization, writing – review and editing.

## Funding

This work was supported by the Noncommunicable Chronic Diseases‐National Science and Technology Major Project (2026ZD0556100, Y.W.), the Hangzhou Leading Innovation and Entrepreneurship Team Project (TD2024015), and the Key R&D Program of Zhejiang (2024SSYS0022, Y.W.).

## Conflicts of Interest

Yongcheng Wang is a co‐founder of M20 Genomics.

## Supporting information




**Supporting File**: advs77013‐sup‐0001‐SuppMat.pdf.

## Data Availability

The data that support the findings of this study are available from the corresponding author upon reasonable request.
