# Supplementary material for: A Microcapsule‐Integrated smRandom‐seq Platform Enables Fixation‐Free Single Microbe RNA Sequencing
Source: Adv Sci (Weinh). 2026 Aug 5;13(60):e77013. doi: 10.1002/advs.77013 (PMC13440202; doi:10.1002/advs.77013)
Supplement: Supplementary file 1 [file ADVS-13-e77013-s001.pdf]

Supporting Information

**A microcapsule-integrated smRandom-seq platform enables fixation-free single microbe RNA sequencing**

*Mengdi Song, Yuting Wang*, Zhaolun Wang, Shunji Zhang, Zhengmin Tang, Yongcheng Wang**

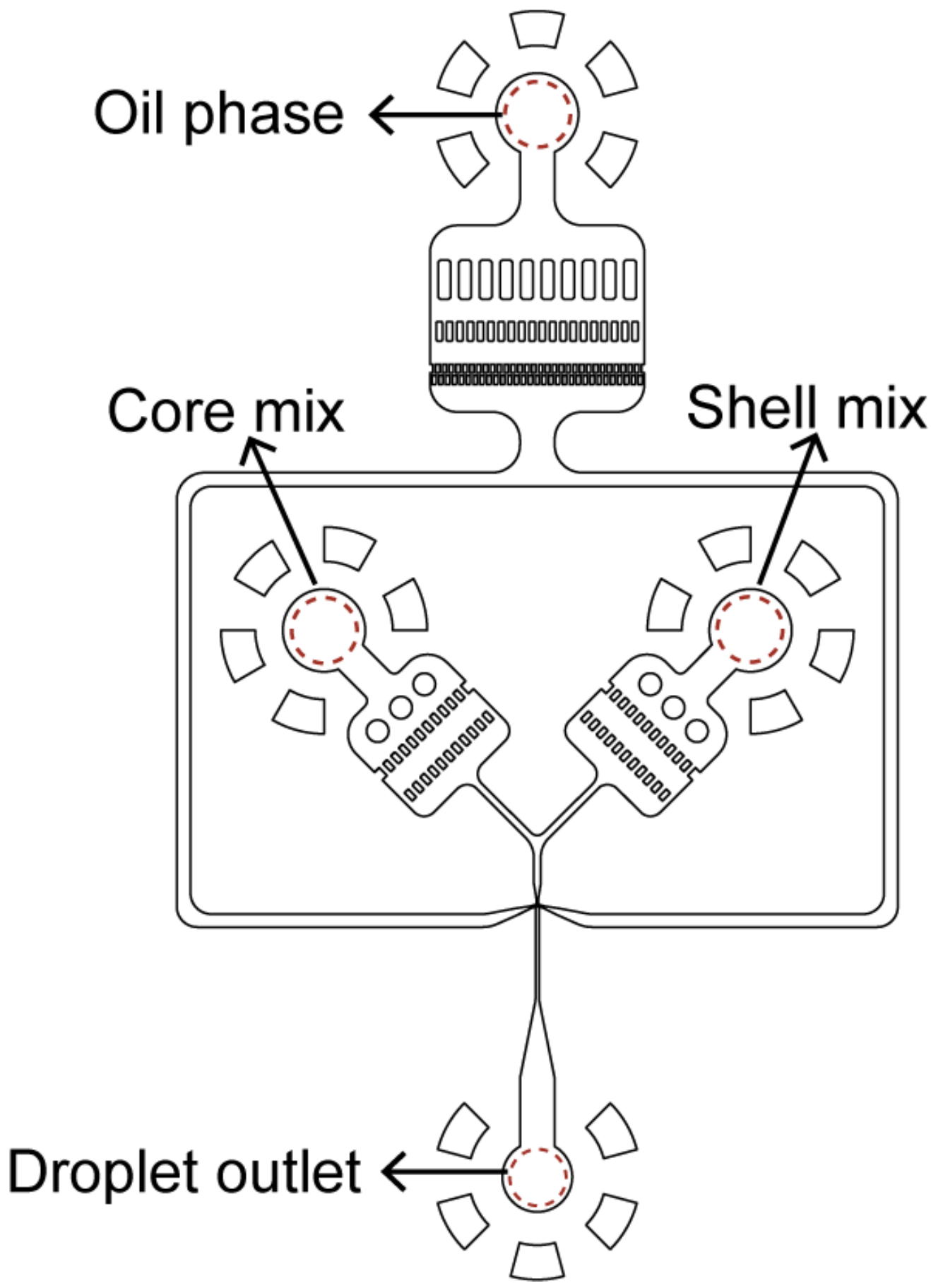


**Figure S1**. **2D design of ATPS capsule generation chip.**

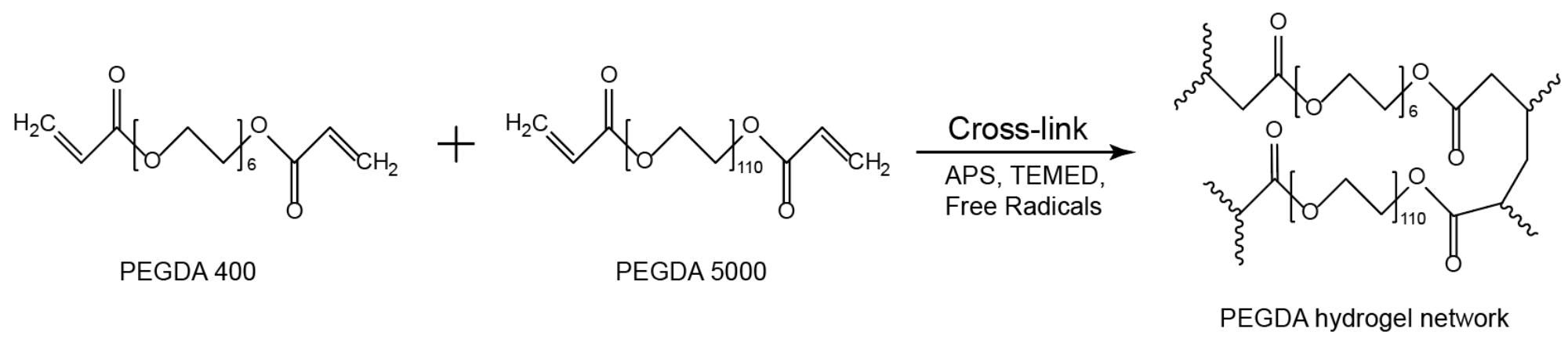


**Figure S2: APS/TEMED-initiated free-radical polymerization of polyethylene glycol diacrylate (PEGDA) 5000 and 400**. Ammonium persulfate (APS) and N,N,N′,N′-tetramethylethylenediamine (TEMED) generate radicals that initiate polymerization of the terminal acrylate groups, resulting in formation of a cross-linked PEGDA hydrogel network.

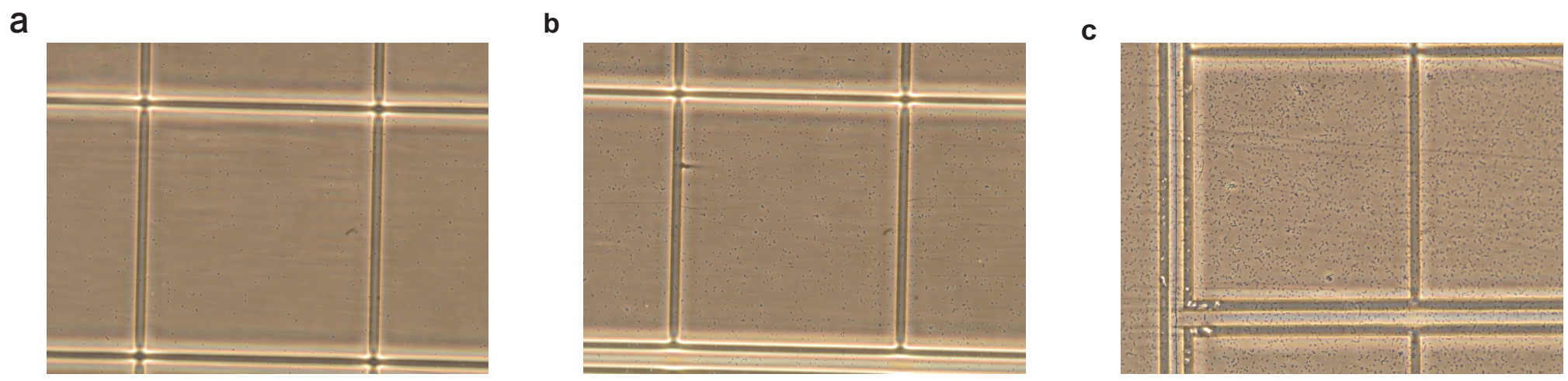


**Figure S3. Culturing test of microbial cells in the presence of F127 and F68 surfactants.** Representative bright field microscopy images of microbial cells cultured under the surfactant conditions used in the workflow, including F127 at 3% and F68 at 0.1%. Images were taken (starting time) at 0 h, 3 h, and 6 h, respectively.

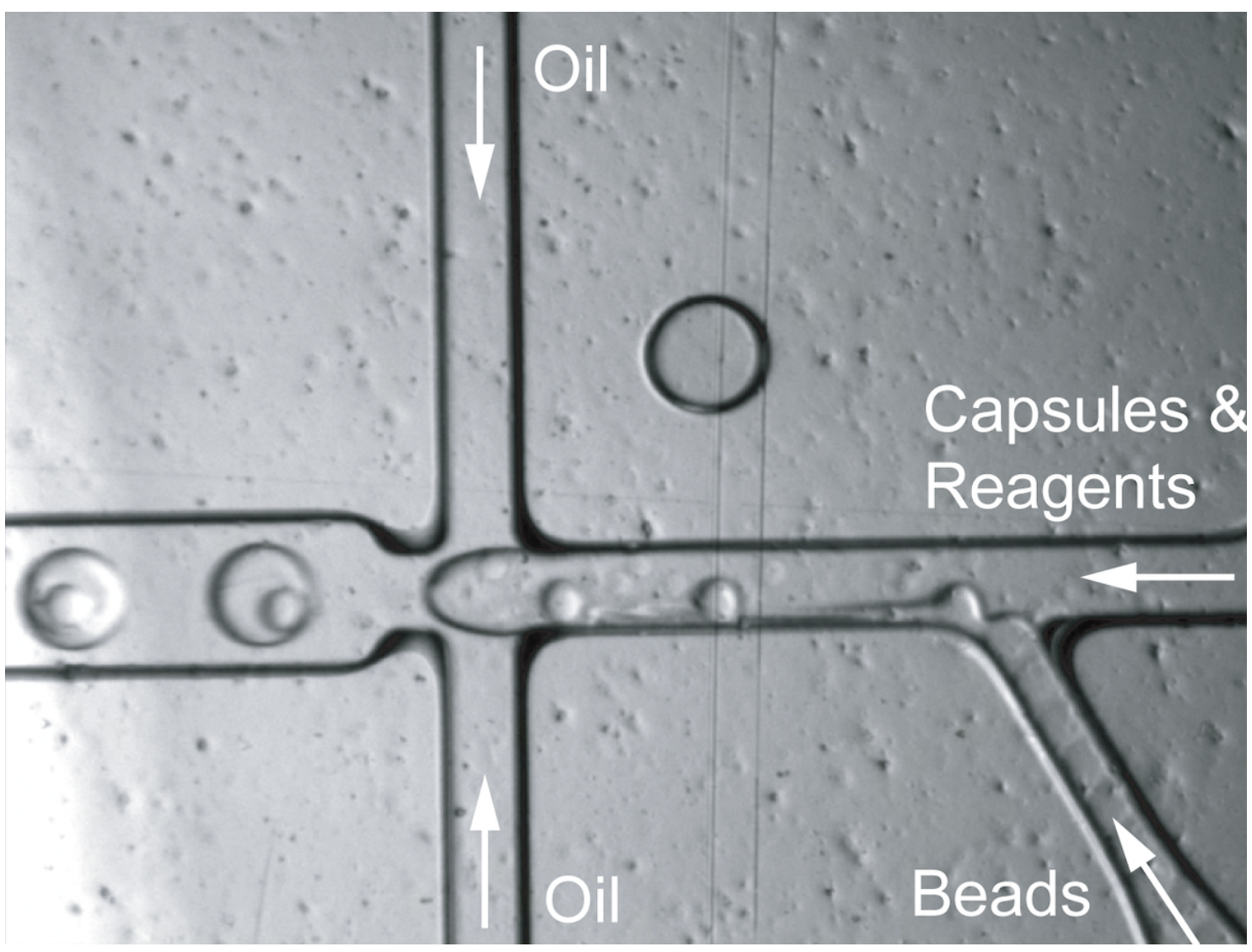

**Figure S4. Co-encapsulation of barcoded-bead and microbe encapsulated microcapsules**

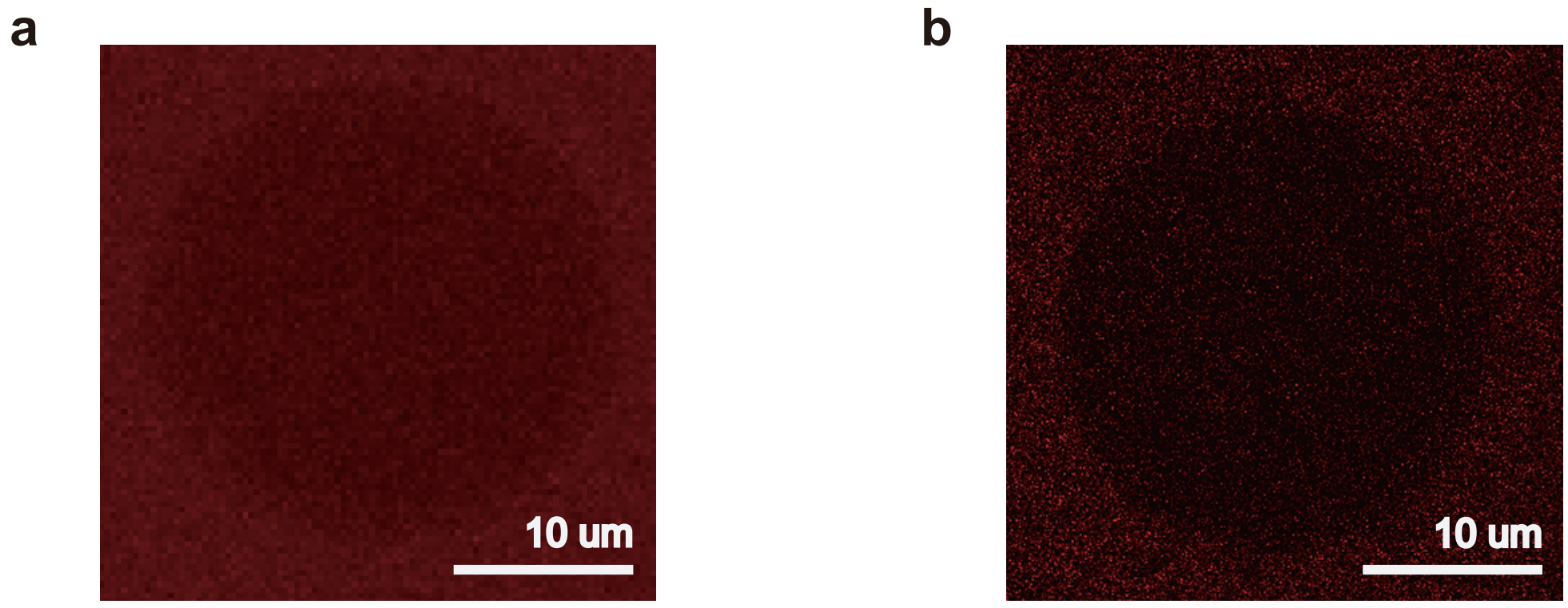


**Figure S5. Functional permeability assessment of PEGDA capsule shells using fluorescent nucleic acid probes. (a)** Representative fluorescence image showing PEGDA capsules after incubation with the 23 bp fluorescent nucleic acid probe. **(b)** Representative fluorescence image showing PEGDA capsules after incubation with the 670 bp fluorescent nucleic acid probe. Scale bar=10 um.

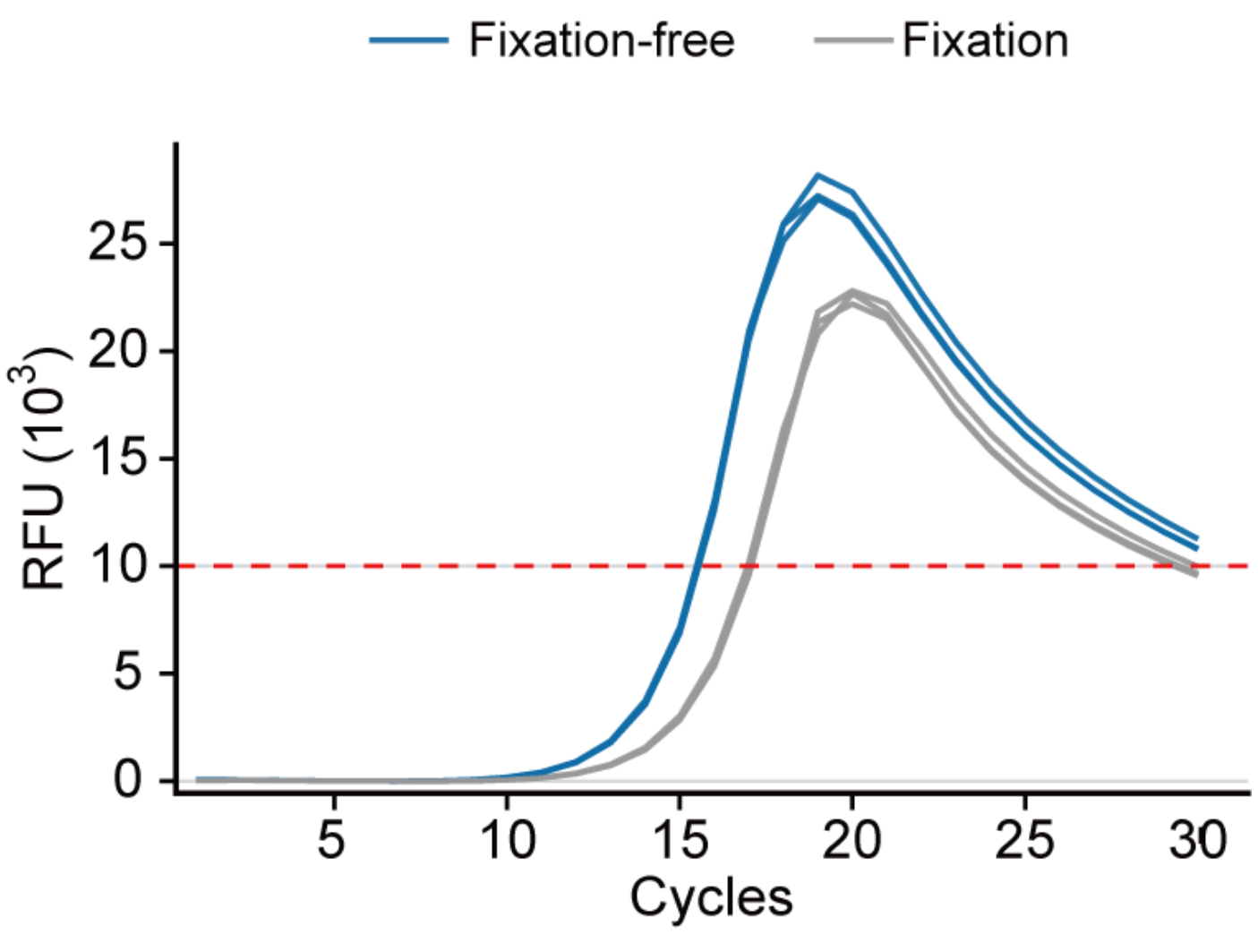


**Figure S6. qPCR qualification curve of fixation-free sample and sample with fixation**

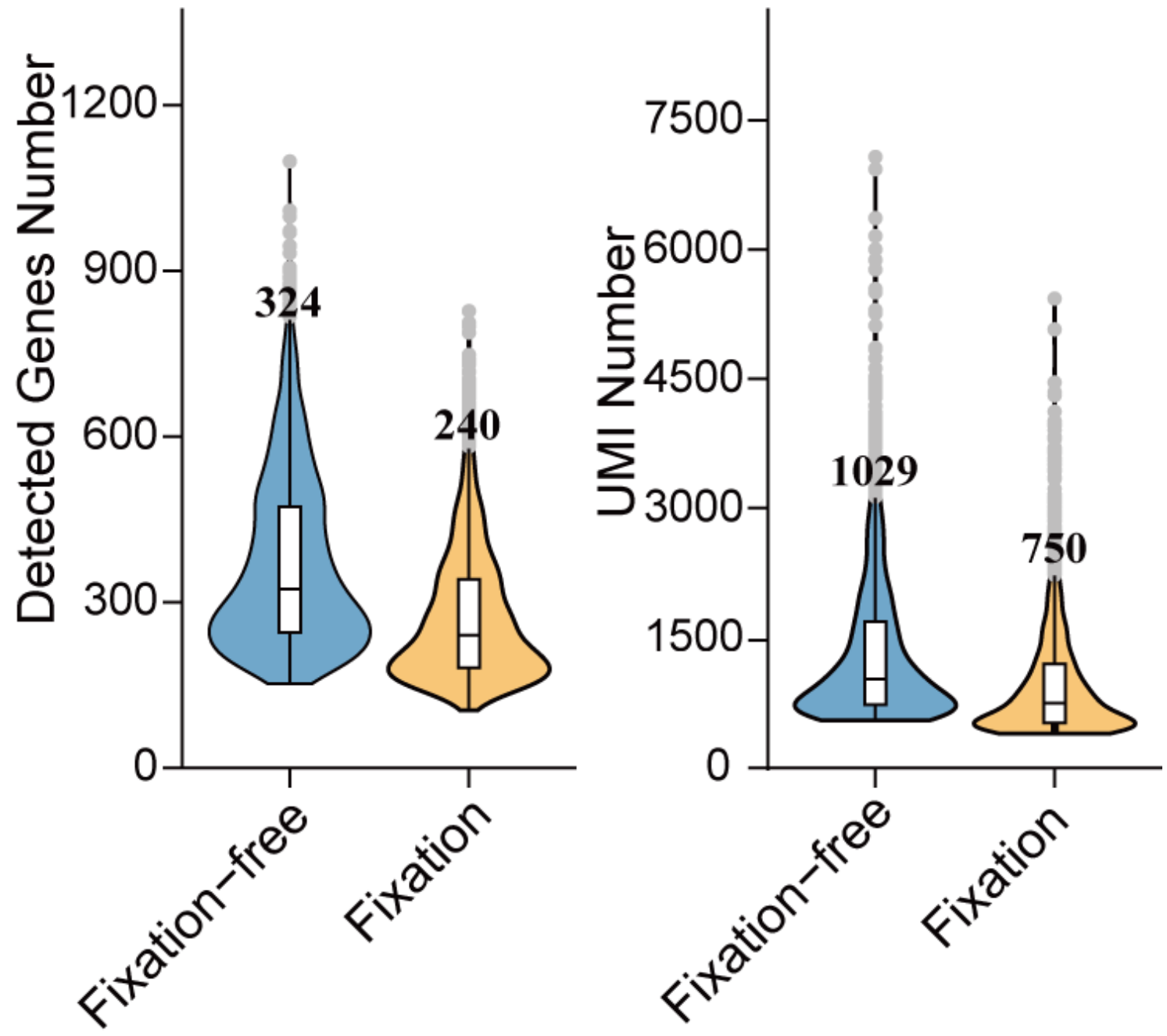


**Figure S7. Violin plots showing distributions of detected gene numbers per barcode under fixation-free and fixed conditions.**

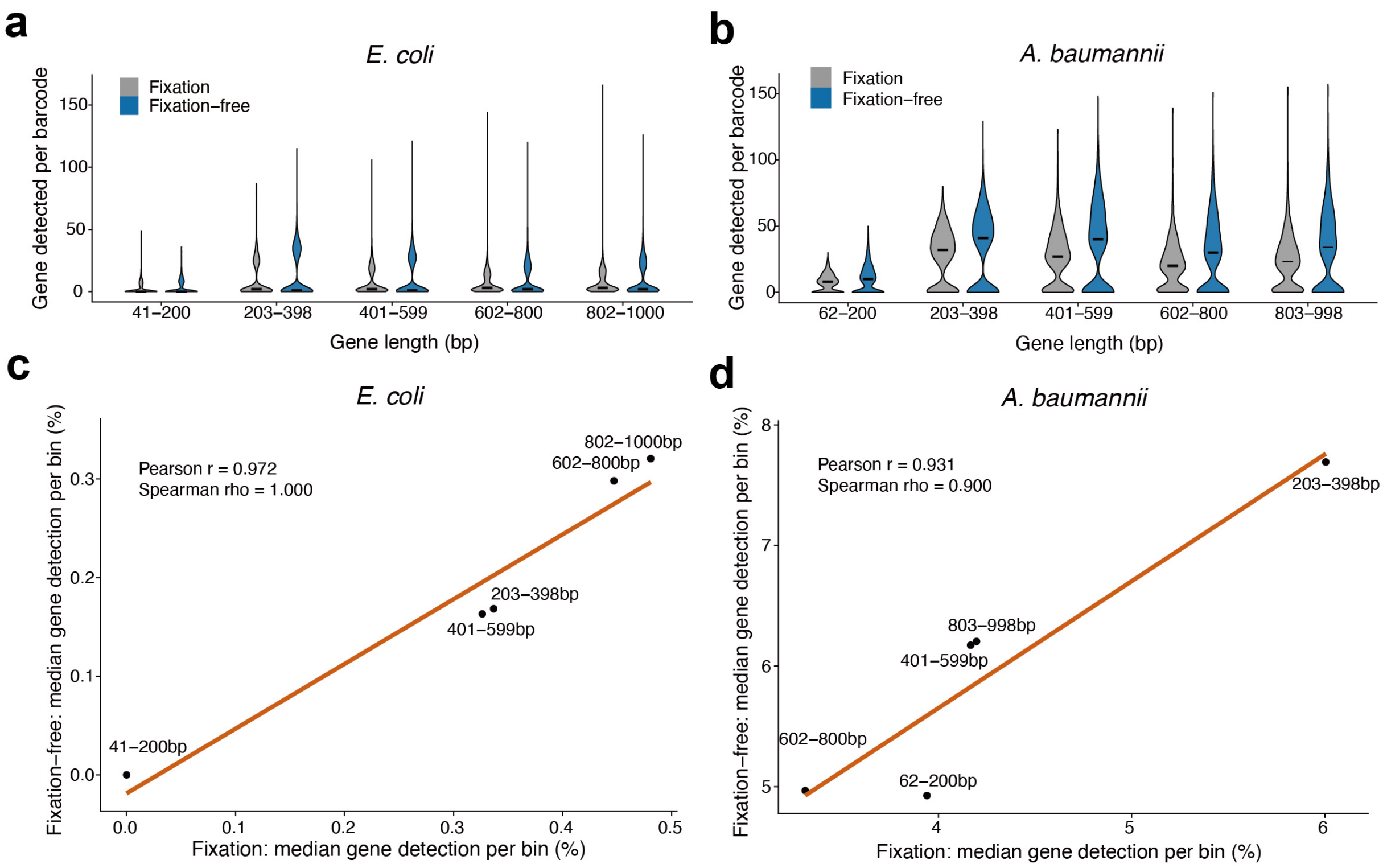


**Figure S8. Gene detection profiles within the 0 to 1,000 bp range in Fixation and Fixation-free workflows.** (a, b) Distribution of detected genes per barcode across gene length bins in the Fixation and Fixation-free workflows for *E. coli* and *A. baumannii*, respectively. Genes were grouped by transcript or gene length, and detection was evaluated per barcode

within each bin. (c, d) Correlation of median gene detection frequency per length bin between Fixation and Fixation free samples for *E. coli* and *A. baumannii*, respectively. Each point represents one gene length bin, and the orange line indicates the fitted linear trend.

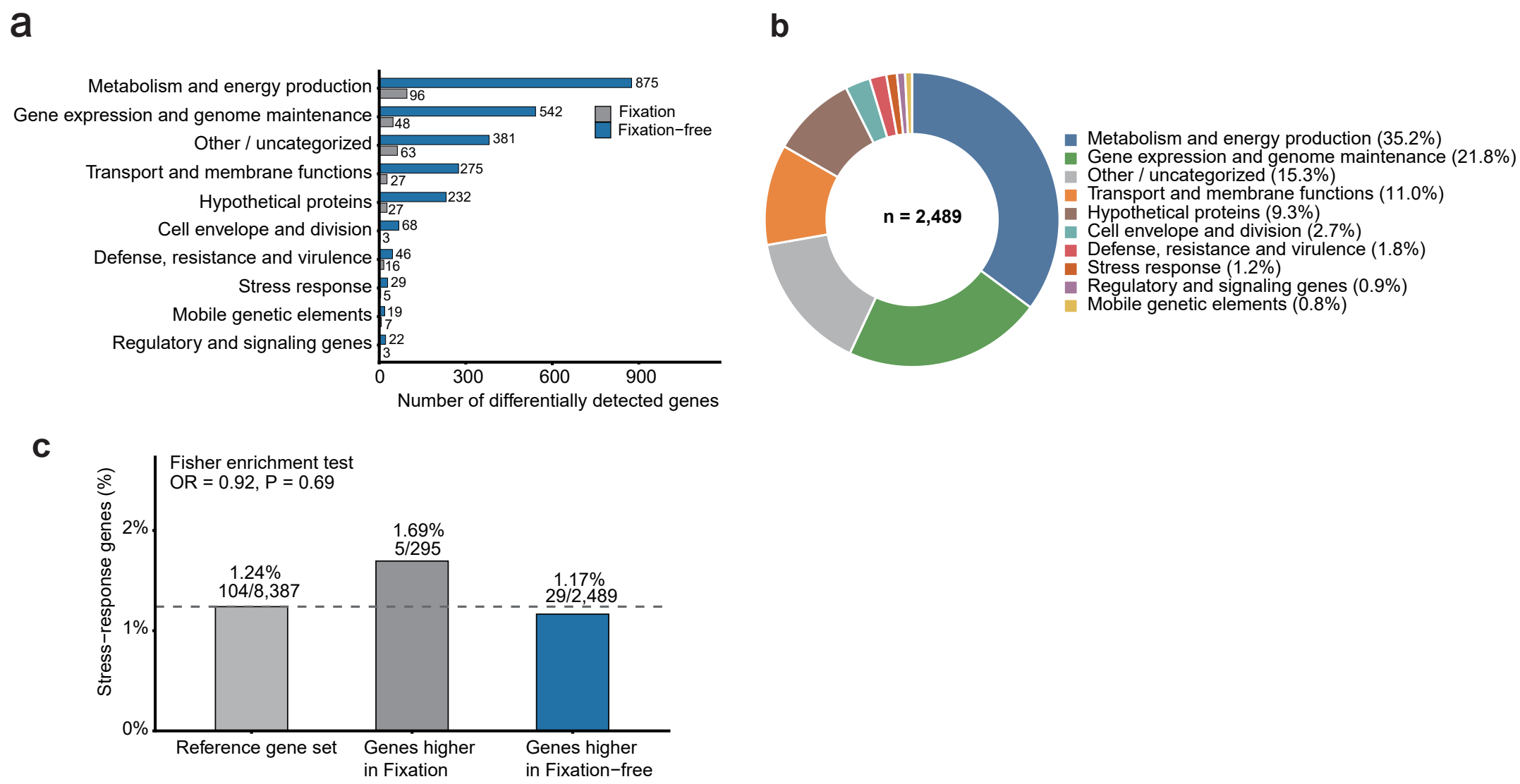


**Figure S9. Functional distribution and stress-response assessment of differentially detected genes between Fixation and Fixation-free workflows.** (a) Bar plot showing the number of differentially detected genes assigned to each functional category, separated by genes higher in the Fixation and genes higher in the Fixation-free. (b) Donut chart showing the functional composition of 2,489 genes preferentially detected in the Fixation free sample. Genes were assigned to major functional categories based on annotation. (c) Bar plot comparing the fraction of annotated stress response genes in the reference gene set, genes higher in Fixation, and genes higher in Fixation free. The dashed horizontal line indicates the stress response gene fraction in the reference gene set and serves as the background level for comparison.
